# An updated data portal for fungal allergens with curated information

**DOI:** 10.6026/97320630015820

**Published:** 2019-12-11

**Authors:** Jun-ichi Onami, Naoki Kobayashi, Maiko Watanabe, Osamu Yamada, Osamu Mizutani, Koji Yokoyama, Takahashi Haruo, Hiroji Chibana, Yoichi Kamata

**Affiliations:** 1 Japan Science and Technology Agency Ringgold standard Institution, National Bioscience Database Center, Tokyo, Chiyoda-ku Japan; 2 National Institute of Health Sciences Ringgold standard Institution, Division of Microbiology Kawasaki, Kanagawa Japan; 3 Azabu University Ringgold standard Institution, School of Life and Environmental Science, Department of Food and Life Science Sagamihara, Kanagawa Japan; 4 National Research Institute of Brewing Ringgold standard institution Hiroshima, Hiroshima Japan; 5 University of the Ryukyus Ringgold standard Institution, Department of Bioscience and Biotechnology Nakagami-gun, Okinawa Japan; 6 6Chiba University Ringgold standard institution, Medical Mycology Research Center Chiba, Chiba Japan; 7 Koshien University Ringgold Standard Institution, School of Nutrition Sciences, Department of Food Design Takarazuka, Hyogo Japan

**Keywords:** Fungal allergens, data

## Abstract

Allergens originating from fungal components abundantly exist in and around human life. We constructed a data portal specific for fungal allergens that includes genomic data from four Aspergillus species used by beverage industries. The fungal database contains the information of nucleotide sequences, which are similar to the coding region of already known allergens in the public database. The database will accelerate allergen identification and prediction in the fungal research field.

## Background

Many materials from plants, arthropods, mammals, and fungi have been reported as allergens that cause an allergic reaction in humans [1]. Among them, fungal allergens abundantly exist in and around modern human life. Some species of fungi called "Koji mold", including Aspergillus oryzae, Aspergillus kawachii, and Aspergillus luchuensis, are used for alcoholic fermentation for sake and shochu produced by traditional Japanese beverage industries. The brewing workers of these beverages are exposed to abundant fungal particles, and a few cases of fungal allergic broncho pulmonary aspergillosis caused by exposure to A. oryzae in a koji brewery have been reported [2].

Many allergen genes were identified in the 1980s [3] accompanying the development of molecular biological techniques. There are several allergen databases that contain allergenic gene sequences and allergenic features. The allergens of A. oryzae and A. fumigatushave oftenbeen reported [4]. In contrast, the allergenicity of A. kawachii and A. luchuensis, which are also used for fermentation in Japanese breweries, have been ambiguous. For finding features of unknown fungal allergens, especially of brewery Aspergilluss pp., we constructed a genomic database specific to fungal allergens of A. kawachii and A. luchuensis. Furthermore, this database also contains the genomic data of A. niger, which is the representative species of the "section Nigri (black aspergilli)" [5]. A. niger is widely used for the production of food ingredients, pharmaceuticals, and industrial enzymes.

## Methodology

**Figure 1:**
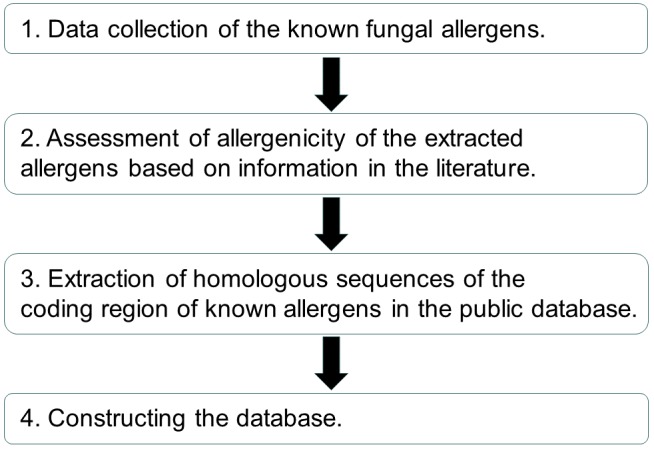
The flowchart for the collection of data and constructing database. We constructed Fungal Allergen Database according to four steps. The constructed datasets were published from this website [9]. A full-text search function via a search box, sorting function in each table, filtering function by source species, and pagination function are implemented by TogoDB [8].

We constructed the database according to the following four steps (Figure 1).

## Data collection of the known fungal allergens:

Fungal allergen data were specifically extracted from the WHO/IUIS allergen nomenclature database (http://www.allergen.org/), which is the storage infrastructure of the entire defined allergen name, standardized by the World Health Organization and the International Union of Immunological Societies [6].

## Assessment of allergenicity of the extracted allergens based on information in the literature:

Extracted WHO/IUIS entries were classified into four ranks based on their allergenicity. Rank 1 indicated that the binding of IgE antibody of the patients to the target recombined or purified allergen protein was confirmed and patients also showed positive reactions to clinical testing (i.e. a skin prick test). Rank 2 indicated that the binding of IgE or IgG antibody of the patients to the recombined or purified allergen protein was confirmed. Rank 3 indicated that the presence of IgE or IgG antibody was confirmed using a clinical test or western blot analysis with roughly extracted allergen protein. Rank 4 indicated that there were no attractive data or ambiguous evidence.

## Extraction of homologous sequences of the coding region of known allergens in the public database:

We performed sequence homology search for similar sequences of the fungal allergen coding region from the genomic data of four Aspergillus spp. (A. niger ATCC 1015, A. niger CBS 513.88, A. luchuensis RIB 2604, A. kawachii NBRC 4308) in the DNA Data Bank of Japan and AspGD database using the BLASTP algorithm [7]. The sequences were classified into four ranks based on e-value score (rank1: the e-value score = 0, rank2: 0 < the e-value score < e-10, rank3: e-10< the e-value score < 1, and rank 4: 1 < the e-value score < 10).

## Constructing the database:

The screened datasets were arranged in a tabular form and entered into the TogoDB database infrastructure [8] to disclose it as a public database. The primary key, label of allergenicity ranking, and a hyperlink to the Uniprot database were added to the database. The database, thus, contains the allergenicity rank and sequence homology rank of each entry for screening "high reliance of allergenicity" and "high sequence conserved regions" of the four Aspergillus genomes.

## Details of database structure and data sorting:

The components of the database were constructed as follows. The allergen data from Ascomycota (82 entries) and Basidiomycota (23 entries) were extracted from the WHO/IUIS allergen nomenclature database, in which there were more than 700 entries total. Among 105 fungal entries, 74 entries contained the allergenicity information. The homologous sequences of the four Aspergillus genomic sequences using the BLASTP algorithm were entered into the database. One hundred and five fungal allergen genes extracted from the WHO/IUIS database were used as the query sequences of BLASTP. The constructed database was opened from the Iwate University server as the "Fungal Allergen Database" [9] with the web interface containing a help page. This database includes 2,486 entries. Each user can screen data by either the e-value rank or the allergenicity rank. Also, the user can download whole datasets via the TogoDB default function [8].

## Conclusion:

Allergens originating from fungal components abundantly exist in and around human life. We constructed a data portal specific for fungal allergens that includes genomic data from four Aspergillus species used by beverage industries. The fungal allergens database contains the information of nucleotide sequences, which are similar to the coding region of already known allergens in the public database. The database will accelerate allergen identification and prediction in the fungal research field.
